# A Bidirectional EF1 Promoter System for Armoring CD19 CAR-T Cells with Secreted Anti-PD1 Antibodies

**DOI:** 10.3390/ijms262311566

**Published:** 2025-11-28

**Authors:** Asmita Khaniya, Nattarika Khuisangeam, Supannikar Tawinwung, Koramit Suppipat, Nattiya Hirankarn

**Affiliations:** 1 Center of Excellence in Immunology and Immune-Mediated Diseases, Department of Microbiology, Faculty of Medicine, Chulalongkorn University, Bangkok 10330, Thailand; ashmita.khaniya1@gmail.com; 2 Center of Excellence in Cellular Immunotherapy, Faculty of Medicine, Chulalongkorn University, Bangkok 10330, Thailand; nattarika.khuisangeam@gmail.com (N.K.); supannikar.t@pharm.chula.ac.th (S.T.); koramit.s@chula.ac.th (K.S.); 3 Medical Microbiology Interdisciplinary Program, Graduate School, Chulalongkorn University, Bangkok 10330, Thailand; 4 Department of Pharmacology and Physiology, Faculty of Pharmaceutical Sciences, Chulalongkorn University, Bangkok 10330, Thailand; 5 Department of Research Affairs, Faculty of Medicine, Chulalongkorn University, Bangkok 10330, Thailand

**Keywords:** bidirectional promoter, CAR-T cells, anti-PD1

## Abstract

Chimeric antigen receptor (CAR) T cell therapy for B cell malignancies is often limited by T cell exhaustion, which is frequently driven by the PD-1/PD-L1 immune checkpoint axis. To overcome this, we developed an “armored” CAR-T cell strategy using a novel bidirectional promoter system. We engineered a single vector to co-express a CD19-specific CAR alongside a secreted anti-PD1 molecule, in either a full-length antibody or a single-chain variable fragment (scFv) format, using the Sleeping Beauty (SB) transposon system. The sequences for the anti-PD1 modules were derived from the clinical antibody nivolumab. Both armored constructs demonstrated robust CAR expression, comparable to or higher than conventional CAR-T cells, and proliferated significantly more than untransfected controls. The engineered cells successfully secreted their anti-PD1 payloads, with the full-length antibody showing more sustained secretion than the scFv. This autocrine blockade resulted in significantly reduced surface PD1 expression on the armored CAR-T cells. Functionally, the anti-PD1-secreting cells exhibited superior cytotoxicity against PD-L1-positive Raji target cells, particularly at low effector-to-target ratios. Critically, in a serial rechallenge assay designed to simulate chronic antigen exposure, both armored CAR-T cell groups showed markedly enhanced proliferation and persistence compared to conventional CAR-T cells, which failed to expand after repeated stimulation. Our findings validate the bidirectional EF1 promoter as an efficient system for generating multi-functional T cells and demonstrate that armoring CAR-T cells with secreted anti-PD1 antibodies is a potent strategy to enhance their persistence and anti-tumor efficacy.

## 1. Introduction

Chimeric antigen receptor (CAR) T cell therapy has transformed the treatment of B cell malignancies. However, long-term efficacy is often limited by T cell exhaustion, a state of dysfunction driven by chronic antigen stimulation and inhibitory signals within the tumor microenvironment [1,2,3]. The Programmed Death-1 (PD-1) pathway is a dominant driver of this process, where PD-L1 on tumor cells engages PD-1 on CAR-T cells to suppress their anti-tumor activity [3,4,5].

To counter this, “armored CAR” strategies have emerged to make T cells resistant to immunosuppression. One promising approach involves engineering CAR-T cells to secrete anti-PD-1 checkpoint inhibitors, creating an autocrine and paracrine blockade directly at the tumor site [6,7,8]. Previous studies have validated this concept, typically using multicistronic vectors that rely on internal ribosome entry sites (IRES) or self-cleaving 2A peptides to co-express the CAR and the secreted molecule from a single transcript. While effective, these elements can sometimes lead to unbalanced protein expression or residual peptide remnants [9,10,11].

Our study introduces a novel and elegant alternative: the use of a bidirectional promoter system. We hypothesized that a single, compact cassette built around the human elongation factor-1 alpha (EF1) promoter could efficiently drive simultaneous expression of the CD19 CAR in one direction and a secreted anti-PD-1 molecule in the opposite (antisense) orientation. This approach could offer a streamlined vector design for robust, dual-gene expression [12].

Here, we validate this bidirectional promoter strategy and use it to directly compare the functional impact of secreting two different formats of an anti-PD1 antibody derived from nivolumab: a full-length IgG antibody and a smaller single-chain variable fragment (scFv). We demonstrate that our system yields functional CAR-T cells that effectively secrete their payload, leading to enhanced cytotoxicity against PD-L1^+^ targets and superior persistence in a long-term rechallenge assay. This work not only confirms the benefit of local anti-PD1 secretion but also establishes the bidirectional EF1 promoter as a powerful and efficient tool for engineering next-generation, multi-functional CAR-T cells.

## 2. Results

### 2.1. CAR Expression in EF1-CAR19- and PD1-Secreting CAR19 T Cells

**Figure 1:**
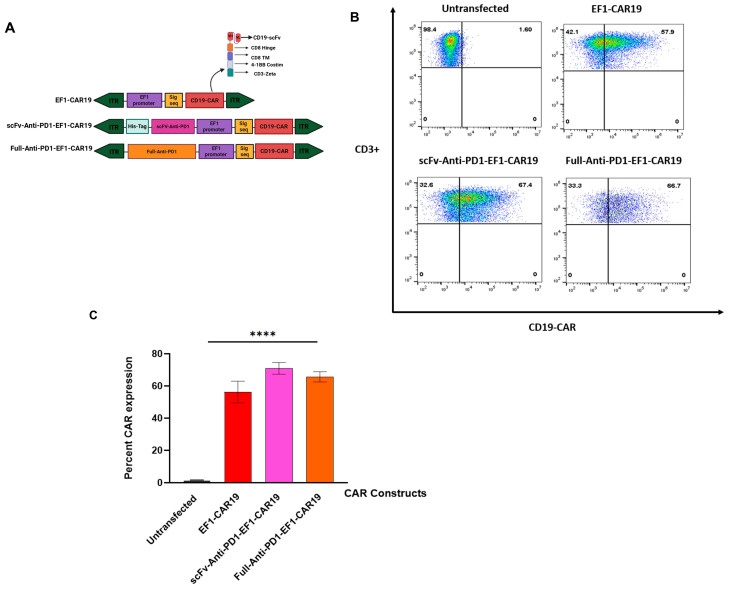
CAR expression in T cells transfected with EF1-driven CAR19 constructs containing PD-1 domains. (**A**) Schematic diagram of with or without scFv and full antibody bearing CD19 CAR, (**B**) Representative flow cytometry plots showing CD3^+^ T cells expressing CAR19, scFv-Anti-PD1-CAR19, or Full-Anti-PD1-CAR19 on day 14 post-transfection. (**C**) CAR expression efficiency across the constructs on day 14 post-transfection. Data represent mean ± SEM from independent experiments (*n* = 3). Significant differences were determined by two-way analysis of variance. Asterisks indicate significant *p* value as follows: **** *p* < 0.0001. Created in BioRender. Khaniya, A. (2025) https://BioRender.com/7qz6fbe (accessed on 4 October 2025).

CAR expression was evaluated on day 14 post-transfection. As expected, untransfected T cells showed negligible CAR expression (1.70 ± 0.13%). In contrast, robust CAR expression was observed in all transfected groups: EF1-CAR19 (56.23 ± 6.73%), scFv-Anti-PD1-EF1-CAR19 (70.97 ± 3.62%), and Full-Anti-PD1-EF1-CAR19 (65.70 ± 3.22%) (Figure 1B,C). Among these, the scFv-Anti-PD1 construct exhibited the highest CAR expression, followed by the full antibody-secreting anti-PD1 construct, both of which were slightly higher than EF1-CAR19 alone. Importantly, the incorporation of PD1-blocking modules, either in scFv or full antibody format, did not impair CAR expression levels, indicating that the additional genetic components did not compromise the efficiency of CAR surface expression.

### 2.2. CAR19 T Cells Exhibit Robust Proliferation Independent of the Addition of PD1 Domains

**Figure 2:**
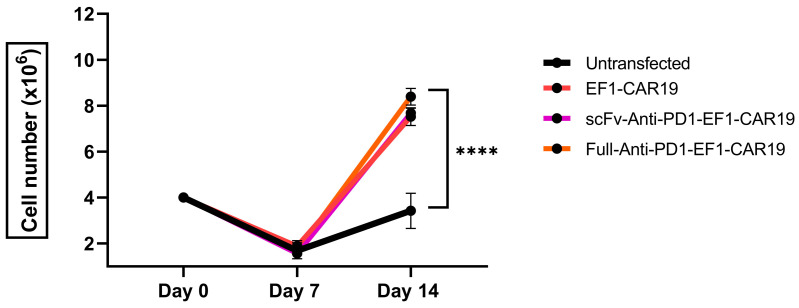
Expansion of CAR T cells with or without PD-1 domains. Cell proliferation was assessed by trypan blue exclusion assay on days 0, 7, and 14 post-transfections. Results are summarized as the mean ± SEM from at least three independent healthy blood donors. Significant differences were determined by two-way analysis of variance. Asterisks indicate significant *p* value as follows: **** *p* < 0.0001.

At day 0, PBMCs were initiated at 4 × 10^6^ cells and expanded over 14 days. CD19-CAR expressing T cells demonstrated markedly greater proliferation compared to untransfected T cells. By day 14, EF1-CAR19, scFv-Anti-PD1-EF1-CAR19, and Full-Anti-PD1-EF1-CAR19 T cells reached 7.5 ± 0.38 × 10^6^ cells, 7.6 ± 0.20× 10^6^ and 8.39 ± 0.36 × 10^6^ respectively. Whereas, untransfected T cells expanded only to 3.42 ± 0.34 × 10^6^ cells (Figure 2). The addition of accessory genes, such as scFv- or full antibody-based PD1 blockers, did not negatively affect CD19-CAR T cell expansion. Both PD1-secreting CD19-CAR constructs exhibited slightly higher proliferation than CD19-CAR alone. Statistical analysis confirmed a highly significant difference between CD19-CAR-transfected groups and the untransfected control, with no significant difference observed between the scFv- and full antibody-secreting PD1 constructs. Although feeder PBMCs were included in both the untransfected control and CD19-CAR-transfected cultures, the enhanced proliferation of the CD19-CAR-transfected cells can be attributed to additional antigen-specific stimulation mediated by the interaction between the CD19-CAR and CD19-expressing feeder cells. In contrast, the untransfected control T cells lacked this specific activation signal and therefore exhibited relatively lower proliferation, despite receiving the same nonspecific stimulation from the feeder PBMCs.

### 2.3. scFv and Full-Length Anti-PD1 CAR T Cells Show Reduced PD1 Expression Compared with Controls

**Figure 3:**
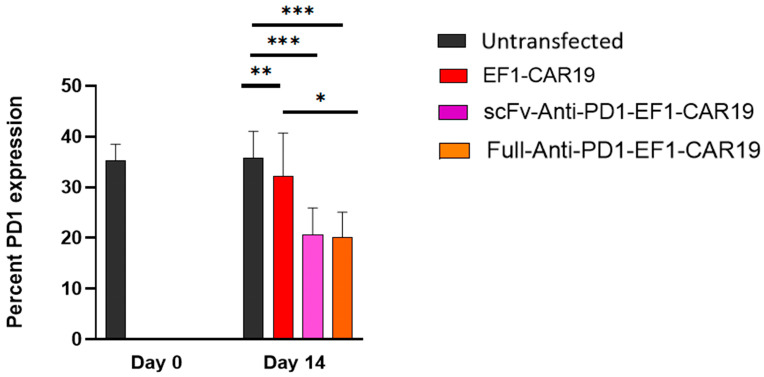
PD-1 expression in CAR T cells with or without anti-PD1 domains. Percent PD-1 expression was measured in CD3^+^ T cells at day 0 and day 14 post-transfection using flow cytometry with anti-PD1 antibody staining. Untransfected cells and EF1-CAR19 served as controls, while scFv-anti-PD1-EF1-CAR19 and Full-anti-PD1-EF1-CAR19 constructs were compared for their effect on PD-1 expression. Data represent mean ± SEM from independent donors (*n* = 3). Significant differences were determined by two-way analysis of variance. Asterisks indicate significant *p* value as follows: * *p* < 0.05, ** *p* < 0.01, *** *p* < 0.001.

We next examined PD1 expression in CD3^+^ T cells on day 14 post-transfection using flow cytometry. At baseline (day 0), PBMCs showed ~35% PD1 expression in CD3^+^ T cells. By day 14, untransfected T cells maintained a similar level of PD1 expression (~36%). EF1-CAR19 T cells exhibited a modest reduction in PD1 expression (~32%), consistent with activation-induced modulation. In contrast, both scFv-Anti-PD1-EF1-CAR19 (~21%) and Full-Anti-PD1-EF1-CAR19 (~20%) T cells demonstrated a significant reduction in PD1 expression compared with untransfected and EF1-CAR19 T cells. These findings indicate that PD1-blocking modules, either in scFv or full antibody format, effectively reduced PD1 expression on the surface of CAR T cells, suggesting functional interference with PD1 signaling without impairing CAR expression or expansion (Figure 3).

### 2.4. Distinct Secretion Kinetics of Full-Length Versus scFv Anti-PD1 Modules in CAR T Cells

**Figure 4:**
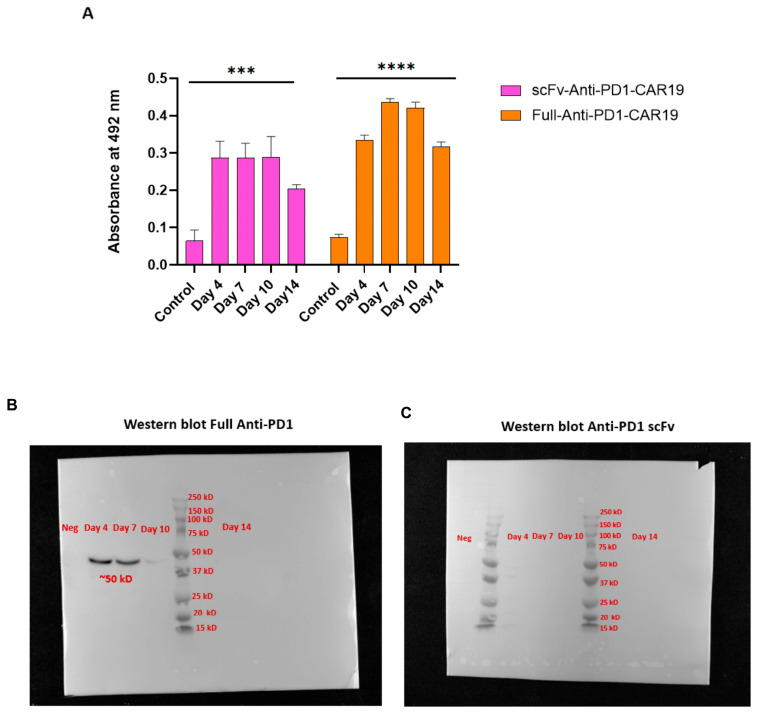
Secretion of anti-PD1 molecules by CAR T cells. (**A**) Supernatants from scFv-anti-PD1-CAR19 and Full-anti-PD1-CAR19 T cells were collected on days 4, 7, 10, and 14 post-transfection and analyzed by ELISA at 492 nm. (**B**) Western blot analysis of supernatants from Full-anti-PD1-CAR19 T cells collected on days 4, 7,10, and 14 post-transfections showed a distinct band at ~50 kDa, confirming stable secretion of the full-length anti-PD1 antibody. (**C**) Western blot analysis of supernatants from scFv-anti-PD1-CAR19 T cells collected on days 4, 7,10, and 14 post-transfections. Data represent mean ± SEM from at least three independent donors. Asterisks indicate significant *p* values as follows: *** *p* < 0.001, **** *p* < 0.0001.

To evaluate the secretion efficiency of the PD1 domains, supernatants from transfected T cells were collected on days 4, 7, 10, and 14 post-transfection and analyzed by ELISA. Both constructs demonstrated measurable secretion of PD1 blocking modules over time, although with distinct kinetics. For the Full-Anti-PD1-CAR19 construct, PD1 antibody secretion increased progressively from day 4 (0.334 ± 0.014) to day 7 (0.437 ± 0.009) and remained stable on day 10 (0.421 ± 0.016) and day 14 (0.317 ± 0.012), indicating sustained but slightly reduced production at the later time point. In contrast, the scFv-Anti-PD1-CAR19 construct exhibited lower secretion levels compared with the full antibody format, with values of (0.287 ± 0.044) on day 4, (0.287 ± 0.039) on day 7, (0.288 ± 0.055) on day 10, and (0.203 ± 0.012) on day 14 Notably, scFv secretion plateaued after day 4 and gradually declined by day 14 (Figure 4A).

Western blot analysis of the culture supernatants confirmed secretion of the full-length anti-PD1 antibody, showing a distinct ~50 kDa band on days 4, 7, and 10, but not on day 14 (Figure 4B). The loss of the day 14 band likely reflects reduced antibody accumulation or degradation of the secreted protein in prolonged culture. In contrast, no visible band corresponding to the scFv was detected at any time point by Western blot, despite clear detection by ELISA (Figure 4C).

### 2.5. Introduction of Anti-PD1 CAR Construct Maintains Subset Distribution and Enriches Memory T Cells

**Figure 5:**
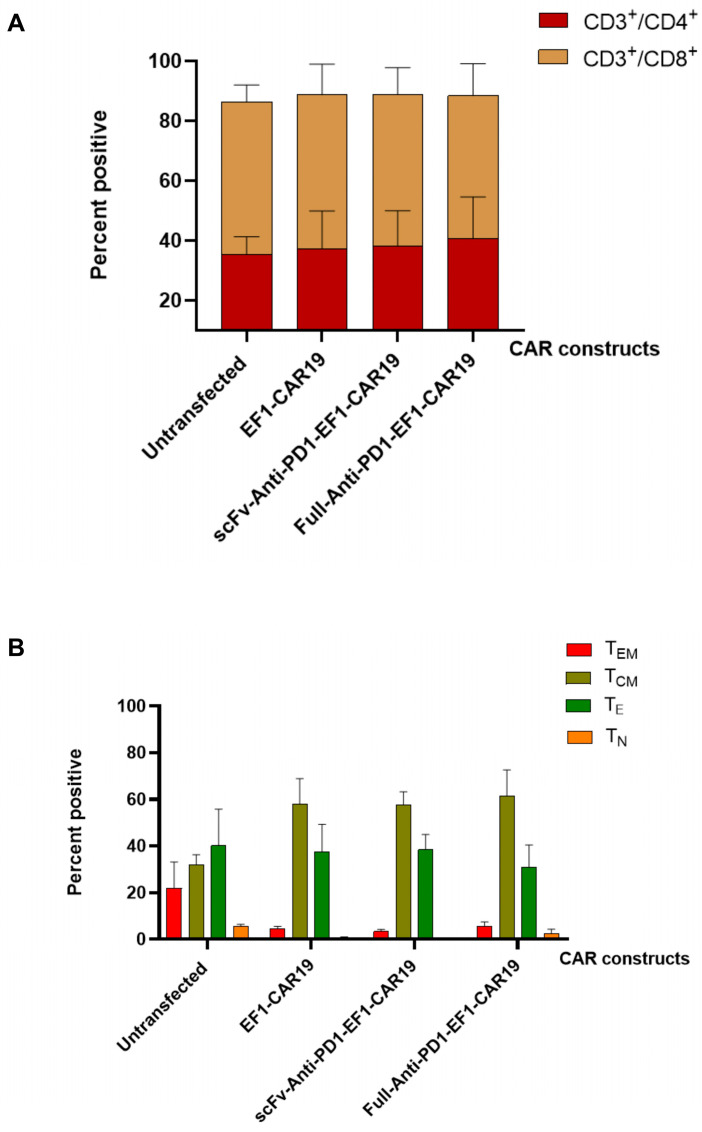
CAR-T cell phenotype at day 14 post-transfection. (**A**) Mean percentages of helper CD3^+^/CD4^+^ T cells and cytotoxic CD3^+^/CD8^+^ T cells on day 14 after transfection. (**B**) Memory phenotype of CD3^+^T cells on day 14 after transfection, showing the percentage of effector memory (T_EM_), central memory (T_CM_), terminal effector (T_E_), and naïve (T_N_) T cells. All results are summarized as the mean ± SEM from at least three independent healthy blood donors.

At day 14 post-transfection, CAR T cells maintained a balanced distribution of CD4^+^ and CD8^+^ subsets, comparable to untransfected controls, with only a slight increase in CD4^+^ cells in the Full-anti-PD1-CAR19 group. This indicates that CAR introduction, including anti-PD1-containing constructs, did not substantially alter the overall CD4^+^/CD8^+^ ratio (Figure 5A). The memory phenotype was determined by the expression of CD45RO and CD62L, as shown in Figure 5B. The memory profile of CAR T cells was clearly shifted compared to untransfected controls. CAR-modified T cells exhibited a strong enrichment of central memory cells, while effector and effector memory populations were reduced. Naïve cells remained a minor fraction across all groups. This shift toward a central memory phenotype suggests that CAR expression favors the development of a population associated with enhanced persistence and long-term functionality.

### 2.6. Cytotoxicity of CAR T Cells Is Preserved in Raji Cells Regardless of PD1-Blocking Module Incorporation

**Figure 6:**
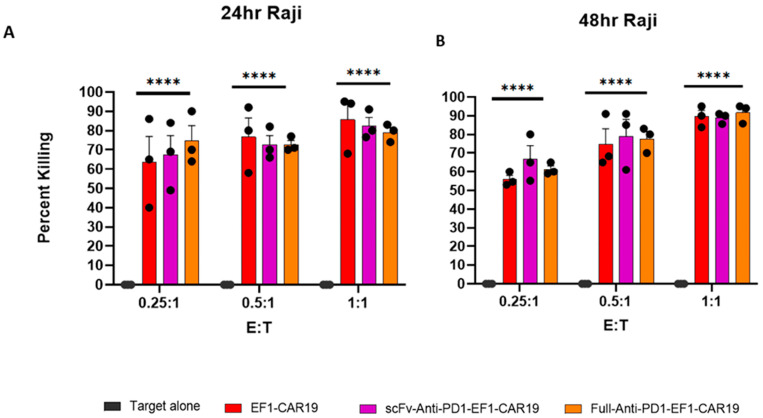
Killing activity of CAR T cells against the Raji target. Cytotoxicity of EF1-CAR19, scFv-Anti-PD1-EF1-CAR19, and Full-Anti-PD1-EF1-CAR19 T cells was evaluated in co-culture with Raji cells at effector to target (E:T) ratios of 0.25:1, 0.5:1, and 1:1. (**A**) 24 h cytotoxicity, (**B**) 48 h cytotoxicity. All results are summarized as the mean ± SEM from at least four independent healthy blood donors. Significant differences were determined by two-way analysis of variance. Asterisks indicate significant *p* values as follows: **** *p* < 0.0001.

The cytotoxic function of CAR T cells was assessed against Raji cells at effector-to-target (E:T) ratios of 0.25:1, 0.5:1, and 1:1 after 24 and 48 h of co-culture. At 24 h, EF1-CAR19 T cells mediated 63.7 ± 13.3% (0.25:1), 76.7 ± 10.0% (0.5:1), and 85.7 ± 8.8% (1:1) killing. scFv-anti-PD1-CAR19 cells achieved 67.3 ± 10.1% (0.25:1), 72.7 ± 4.8% (0.5:1), and 82.5 ± 4.3% (1:1) killing, while Full-anti-PD1-CAR19 cells showed 74.7 ± 7.9% (0.25:1), 72.7 ± 2.2% (0.5:1), and 79.0 ± 2.6% (1:1) killing (Figure 6A). At 48 h, overall cytotoxicity further increased. EF1-CAR19 mediated 55.9 ± 2.1% (0.25:1), 74.8 ± 8.2% (0.5:1), and 89.6 ± 3.2% (1:1) killing. scFv-anti-PD1-CAR19 cells reached 66.7 ± 7.2% (0.25:1), 79.0 ± 9.2% (0.5:1), and 88.8 ± 1.7% (1:1) killing, while Full-anti-PD1-CAR19 achieved 61.3 ± 1.9% (0.25:1), 77.7 ± 3.9% (0.5:1), and 91.6 ± 2.9% (1:1) killing (Figure 6B). Across all ratios, cytotoxic activity remained comparable between EF1-CAR19 and PD1-modified constructs.

### 2.7. Full- and scFv-Anti-PD1 CAR T Cells Show Improved Early Killing of Raji-PDL1 Cells Compared with EF1-CAR19

**Figure 7:**
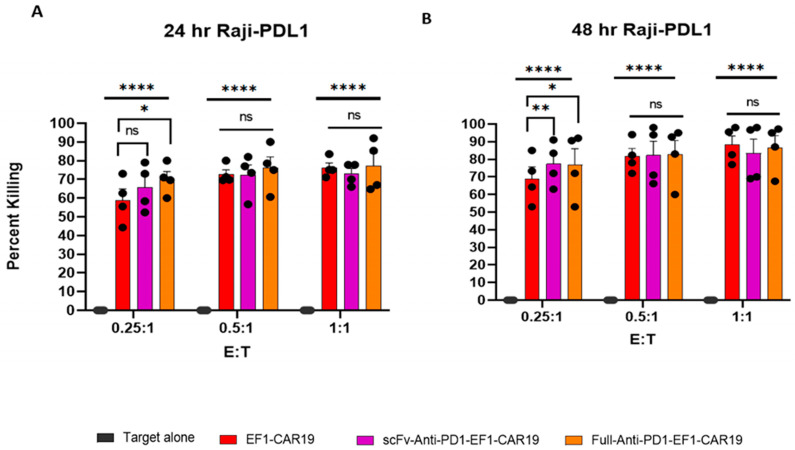
Killing activity of CAR T cells against Raji-PDL1 target. Cytotoxicity of EF1-CAR19, scFv-Anti-PD1-EF1-CAR19, and Full-Anti-PD1-EF1-CAR19 T cells was evaluated in co-culture with Raji-PDL1 cells at effector-to-target (E:T) ratios of 0.25:1, 0.5:1, and 1:1. (**A**) 24 h cytotoxicity, (**B**) 48 h cytotoxicity. All results are summarized as the mean ± SEM from at least four independent healthy blood donors. Significant differences were determined by two-way analysis of variance. Asterisks indicate significant *p* values as follows: * *p* < 0.05, ** *p* < 0.01, **** *p* < 0.0001 and ns, non-significant.

The cytotoxic function of CAR T cells was evaluated against the Raji-PDL1 target at effector to target (E:T) ratios of 0.25:1, 0.5:1, and 1:1 after 24 and 48 h of co-culture. At 24 h, EF1-CAR19 cells mediated 58.9 ± 6.0%, 72.6 ± 2.5%, and 76.2 ± 2.6% killing at 0.25:1, 0.5:1, and 1:1 ratios, respectively. scFv-Anti-PD1-CAR19 cells showed 65.7 ± 6.2%, 72.3 ± 5.5%, and 72.9 ± 2.9% killing, while Full-Anti-PD1-CAR19 cells exhibited the highest activity with 70.2 ± 4.1%, 76.0 ± 6.1%, and 77.3 ± 6.7%, respectively. At the lowest E:T ratio (0.25:1), only Full-Anti-PD1-CAR19 displayed significantly enhanced cytotoxicity compared to EF1-CAR19, whereas no significant differences were observed at higher ratios (Figure 7A and Supplementary Figure S2). At 48 h, killing efficiency further increased across all groups. EF1-CAR19 cells mediated 68.9 ± 6.8%, 81.6 ± 4.7%, and 88.3 ± 5.1% killing at 0.25:1, 0.5:1, and 1:1 ratios, respectively. scFv-Anti-PD1-CAR19 cells reached 77.4 ± 6.2%, 82.3 ± 8.0%, and 83.4 ± 8.1%, while Full-Anti-PD1-CAR19 cells exhibited 76.9 ± 9.2%, 82.7 ± 8.0%, and 86.7 ± 6.8% killing at the respective ratios. At the 0.25:1 ratio, both scFv-Anti-PD1-CAR19 and Full-Anti-PD1-CAR19 achieved significantly greater killing compared to EF1-CAR19, while no differences were observed among groups at higher ratios (Figure 7B and Supplementary Figure S2).

Together, these findings demonstrate that full-length anti-PD1 secretion enhances cytotoxicity at early time points under limiting effector-to-target conditions, while by 48 h, both scFv- and full-length anti-PD1–modified CAR T cells confer improved killing relative to conventional CAR T cells.

### 2.8. Rechallenge Assay Reveals Enhanced Proliferation of PD1 Antibody-Bearing CAR T Cells

**Figure 8:**
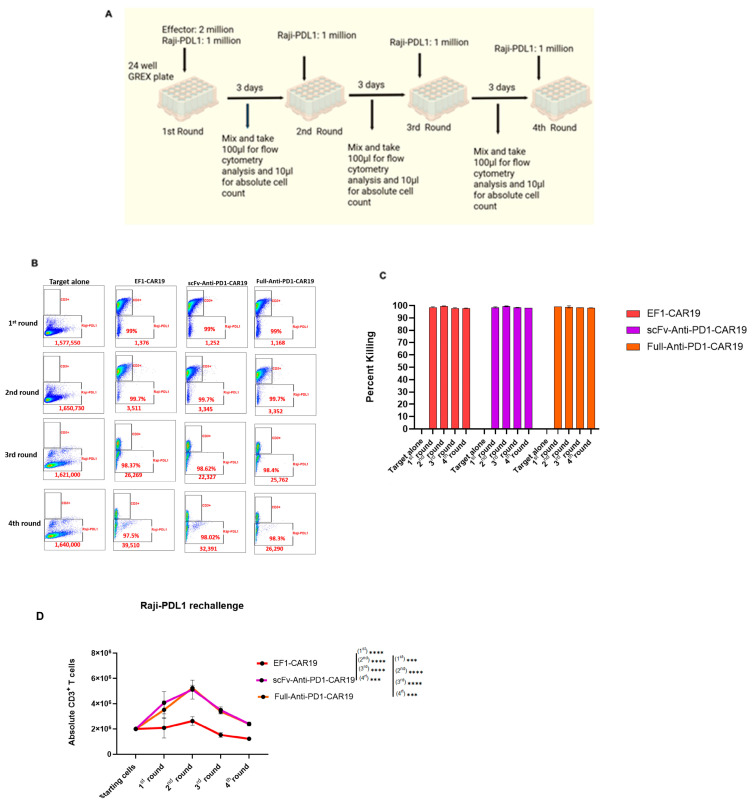
Rechallenge assay of CAR T cells with Raji-PDL1 target. (**A**) Schematic of the rechallenge experimental design. CAR T cells were co-cultured with Raji-PDL1 cells at a 2:1 effector-to-target ratio in 24-well GREX plates. (**B**) Representative flow cytometry plots showing CD3^+^ T cells and Raji-PDL1 target cells across sequential rechallenges. (**C**) Cytotoxicity after each round of rechallenge assay (**D**). Absolute numbers of CD3^+^ T cells during four rounds of rechallenge. All results are summarized as the mean ± SEM from two independent healthy blood donors. Significant differences were determined by two-way analysis of variance. Asterisks indicate significant *p* values as follows: *** *p* < 0.001, **** *p* < 0.0001. Created in BioRender. Khaniya, A. (2025) https://BioRender.com/8hjh44y (accessed on 4 October 2025).

To evaluate the proliferative potential and persistence of CAR T cells in the presence of repeated antigen stimulation, a rechallenge assay was performed using Raji-PDL1 target cells. Effector cells were co-cultured with Raji-PDL1 at a 2:1 ratio in 24 24-well GREX plate and subjected to four rounds of rechallenge every three days. At each round, cells were harvested for absolute counts and flow cytometric analysis (Figure 8A).

Across four sequential stimulations, all three groups, EF1-CAR19, scFv-Anti-PD1-CAR19, and Full-Anti-PD1-CAR19 cells demonstrated robust tumor killing activity (Figure 8B,C). However, scFv-Anti-PD1-CAR19 and Full-Anti-PD1-CAR19 T cells exhibited markedly superior proliferative capacity compared to EF1-CAR19 controls. Both PD1-modified CAR T cell groups expanded more vigorously after the first stimulation, reaching their peak during the second round, with cell numbers exceeding those of EF1-CAR19. In contrast, EF1-CAR19 T cells showed limited expansion, with absolute cell numbers declining substantially after the second round. By the third and fourth rounds, both scFv- and full anti-PD1 modified CAR T cells maintained significantly higher CD3^+^ T cell numbers relative to EF1-CAR19, indicating enhanced persistence under repeated antigen exposure (Figure 8D).

## 3. Discussion

In this study, we successfully engineered CD19-specific CAR-T cells to resist PD1-mediated immunosuppression by secreting anti-PD1 antibodies. The central novelty of our work lies in the successful application of a bidirectional EF1 promoter system to achieve this armoring effect [12]. Our results validate this engineering strategy as a robust and efficient method for co-expressing a CAR and a therapeutic secreted protein from a single, compact genetic cassette. The data confirm that the bidirectional promoter effectively drives high-level expression of both transgenes. We observed strong surface CAR expression and significant secretion of both the full-length antibody and the scFv format. This demonstrates that the promoter functions efficiently in both orientations without apparent steric hindrance or promoter interference, addressing a potential challenge in multi-gene vector design. This streamlined approach avoids the use of IRES or 2A peptides for co-expression, offering a compelling alternative for creating multi-functional cell therapies. Functionally, this engineering strategy translated into a clear therapeutic advantage [12,13,14,15].

Notably, a discrepancy was observed in the detection of the secreted anti-PD1 modules, indicating possible variations in their stability or detectability across assays. While the full-length antibody was readily detected by both ELISA and Western blot, the scFv module was only detectable by ELISA. This difference could possibly reflect a combination of factors related to protein stability and assay sensitivity. The scFv, a small fragment (~25–30 kDa) lacking the stabilizing glycoprotein structure of a full-length IgG, may be more susceptible to proteolytic degradation in culture supernatants. This interpretation is supported by the ELISA kinetics, where scFv secretion appeared to plateau after day 4, suggesting a possible steady state in which secretion is balanced by degradation. In contrast, the more stable full-length antibody seemed to continue accumulating over time [16,17]. As a result, it is possible that the low, steady-state concentration of scFv was sufficient for detection by the highly sensitive ELISA but fell below the detection threshold of the Western blot analysis. Although Ni-Sepharose enrichment could improve detectability, this was not performed within the scope of the current study. We acknowledge this methodological limitation and plan to incorporate affinity-based concentration in future optimization efforts [18].

Functionally, autocrine PD-1 blockade may partially mitigate CAR-T cell exhaustion mediated by the PD-1/PD-L1 pathway, as reflected by the enhanced proliferation and persistence observed in our multi-round rechallenge assay. However, other exhaustion mechanisms may also contribute to this effect. A limitation of our study is that we did not assess additional exhaustion markers such as LAG-3, TIM-3, or KLRG-1, which could provide a more comprehensive understanding of the exhaustion landscape. Future studies will include these markers to further elucidate the broader mechanisms underlying CAR-T cell exhaustion [10,15,16]. This is a critical finding, as CAR-T cell persistence is a known correlate of long-term clinical remission. Furthermore, the armored CAR-T cells displayed a favorable enrichment of the central memory (TCM) phenotype, a cell population associated with longevity and potent recall responses. This suggests that continuous relief from PD-1 signaling not only boosts immediate effector function but also helps maintain a healthier, more durable T cell population [3,17,18].

Although in vivo validation remains to be performed, the primary objective of this study was to establish a rigorous in vitro proof-of-concept for the bidirectional promoter strategy and to conduct a direct functional comparison of the full-length antibody and scFv formats. Our comprehensive cellular assays, particularly the demanding rechallenge experiment, provide a strong foundation by demonstrating the feasibility, mechanism, and functional benefit of our constructs. These promising results establish the necessary groundwork and provide a compelling rationale for subsequent, resource-intensive preclinical evaluation in animal models to assess in vivo efficacy and safety.

In conclusion, our study validates a bidirectional promoter system as a powerful tool for developing armored CAR-T cells. We show that using this system to drive local secretion of anti-PD1 antibodies, particularly in the full-length format, which showed more robust secretion, is an effective strategy to enhance CAR-T cell persistence and anti-tumor activity. This work contributes a valuable and efficient vector design strategy for the ongoing effort to build safer and more effective cell-based immunotherapies.

## 4. Materials and Methods

### 4.1. Primary Cells and Cell Lines

Raji and Raji-PDL1 overexpressed (human Burkitt lymphoma cell line) cells were maintained in RPMI 1640 medium supplemented with Glutamax (Thermo Fisher Scientific, Waltham, MA, USA), penicillin (100 U/mL) streptomycin (100 µg/mL) and 10% heat-inactivated fetal bovine serum (FBS) (Thermo Fisher Scientific, Waltham, MA, USA) at 37 °C with 5% CO_2_. PBMCs were obtained from healthy blood donors with informed consent, and all protocols were approved and conducted according to the rules and regulations of the Institutional Review Board of the Faculty of Medicine, Chulalongkorn University (IRB NO. 0203/66).

### 4.2. Generation of Full-Anti-PD1-EF1-CAR19 and scFv-Anti-PD1-EF1-CAR19 in Sleeping Beauty (SB) Transposon System

Full-Anti-PD1 and scFv-Anti-PD1 antibody sequences were derived from the published Nivolumab (Opdivo) heavy and light chain variable region sequences available in the DrugBank database (DrugBank Accession Number: DB09035; https://go.drugbank.com/drugs/DB09035 (25 October 2025)). The scFv was designed by linking the VH and VL regions with a (G_4_S)_3_ linker to form a single-chain variable fragment, and a C-terminal 6×His tag (5′-CATCATCATCATCACCAT-3′) was added downstream of the scFv coding region. For the expression of a full-length anti-PD1 antibody, the heavy chain (HC) and light chain (LC) coding sequences were linked via a T2A self-cleaving peptide sequence under the EF1 promoter. Furin recognition sites and a short GSG (G_3_S) linker were added to improve cleavage efficiency and reduce residual peptide remnants. To facilitate efficient expression in human cells, codon optimization was performed using Gene Designer 2.0. The complete scFv-His sequence, along with the full-length anti-PD1 sequence, was synthesized by GenScript, 860 Centennial Ave, Piscataway, NJ 08854, USA. Both synthesized inserts were PCR-amplified using specific forward and reverse primers (Full-Anti-PD1-FP: 5′-caccgcatccccacatgttattacattcgcctcggttga-3′; Full-Anti-PD1-RP: 5′-cgggcaccggagcgcatgtcgccaccatgggatggt-3′; scFv-Anti-PD1-FP: 5′-caccgcatccccagcatgttaatggtgatgatgatgatggcg-3′; scFv-Anti-PD1-RP: 5′-cgggcaccggagcgcatgattcgccaccatggaatttgg-3′) containing homologous overhangs approximately ~20 bp correspondence to the EF1-CAR19 backbone. To generate EF1-CAR19, the EF1 promoter was amplified from the pSBbi-RP plasmid (gift from Eric Kowarz) [19], then CD19 CAR (1582 bp) FMC63 anti-CD19-scFv, the human CD8 hinge domain, the human CD8 transmembrane (TM), 4-1BB intracellular costimulatory domain, and the CD3ζ activation domain were cloned into the pSBbi-RP backbone. EF1-CAR19 plasmid was then linearized by SphI digestion, and either the full-length Anti-PD1 or scFv Anti-PD1 insert was cloned upstream of the EF1 promoter in the antisense orientation using Gibson assembly (Gibson Assembly^®®^ Protocol: E5510) (Figure 1A and Supplementary Figure S1)

Following cloning, bacterial colonies were screened by restriction digestion and Sanger sequencing to confirm the correct constructs. A verified clone was inoculated into 10 mL of LB broth supplemented with ampicillin and cultured until OD_600_ reached 0.5. The entire 10 mL culture was then transferred into 250 mL LB broth with ampicillin and incubated overnight. Using the standard conversion of OD_600_ = 0.5 ≈ 5 × 10^8^ cells/mL, the 250 mL culture contained approximately 1.25 × 10^11^ bacterial cells. Plasmid DNA was subsequently isolated using an endotoxin-free Maxiprep kit (Catalogue #12362) according to the manufacturer’s instructions.

### 4.3. Isolation of Peripheral Blood Mononuclear Cells (PBMCs)

Peripheral blood mononuclear cells (PBMCs) were obtained from healthy blood donors of both sexes. Written informed consent was obtained from all participants, and the study protocol was approved by the Institutional Review Board of the Faculty of Medicine, Chulalongkorn University (IRB no. 0203/66). PBMC isolation was performed using Ficoll-Paque™ Premium (GE Healthcare, Bio-Sciences AB, Vendevägen 89, 182 32 Danderyd, Sweden) density gradient centrifugation according to the manufacturer’s instructions.

Briefly, whole blood was diluted 1:1 with 1× PBS, and 20 mL of diluted blood was gently layered onto 15 mL of Ficoll-Paque in a 50 mL conical tube. Samples were centrifuged at 400× *g* for 30 min at room temperature with no brake. The mononuclear cell layer at the plasma–Ficoll interface was carefully collected and transferred into a new 50 mL tube, avoiding contamination with other fractions. Cells were then washed twice with 1× PBS by centrifugation at 400× *g* for 10 min. The resulting PBMC pellet was resuspended in culture medium, and viable cells were counted using a Neubauer chamber.

### 4.4. Generation of CAR T Cells

Fresh peripheral blood mononuclear cells (PBMCs) were isolated from healthy donor blood using Ficoll-Paque™ Premium (GE Healthcare, Bio-Sciences AB, Uppsala, Sweden) through density gradient centrifugation. Plasmids were purified from NEB^®®^ 5-alpha Competent *E. coli* using endotoxin-free Qiagen plasmid maxi kits (Hilden, Germany). PBMCs (4 × 10^6^) were resuspended in P3 Primary Cell 4D-Nucleofector™ X Kit reagent (Lonza, Basel, Switzerland) together with CAR constructs (SB transposon; 2 µg) and pCMV-CAT (SB transposase; 0.4 µg) (kind gift from Zsuzsanna Izsvak) [20] at a 5:1 ratio in a final volume of 20 µL. Electroporation was performed using the 4D-Nucleofector™, after which cells were immediately incubated in TexMACs™ medium (Miltenyi Biotec, Bergisch Gladbach, Germany) supplemented with 5% FBS, 10 ng/mL IL-7, and 5 ng/mL IL-15 at 37 °C with 5% CO_2_ for 10 min.

In parallel, feeder PBMCs (10–15 × 10^6^) were irradiated with 25 Gy and resuspended in TexMACs™ medium containing 5% FBS, 10 ng/mL IL-7, and 5 ng/mL IL-15. Electroporated cells were cocultured with irradiated feeder cells in 48-well plates and maintained for 14 days. Media was refreshed on day 4 and day 7 with fresh TexMACs™ medium supplemented with 5% FBS, 10 ng/mL IL-7, and 5 ng/mL IL-15.

### 4.5. Cell Expansion and Phenotypic Analysis

Cell expansion and viability were assessed on days 0, 7, and 14 after transfection using the trypan blue exclusion assay. For flow cytometric analysis, CAR T cells (2 × 10^5^ cells per test) were stained with the indicated antibodies at 4 °C for 15 min. Samples were acquired on a MACSQuant Analyzer 10 Flow Cytometer (Miltenyi Biotec, Bergisch Gladbach, Germany), and data were analyzed using FlowJo software (version 10.7.1). Lymphocytes were first gated on forward and side scatter (FSC/SSC), followed by selection of CD3^+^ T cells.

Transfection efficiency was determined using a PE-labeled idiotypic monoclonal antibody specific for FMC63 scFv (ACROBiosystems, Newark, DE, USA). T cell subsets were analyzed with CD3-PE (OKT3), CD8-APC (SK1) (BD Biosciences, San Jose, CA, USA), and CD4-FITC (BioLegend, San Diego, CA, USA). Memory subsets were evaluated using CD3-PerCP (UCHT1), CD45RO-VioGreen (UCHL1), and CD62L-VioBlue (DREG-56) (BD Biosciences). PD-1 expression was assessed with CD3-APC (OKT3) and CD279 (PD-1)-FITC (BioLegend, San Diego, CA, USA).

### 4.6. Detection of Full-Anti-PD1 and scFv-Anti-PD1 by ELISA

To evaluate the binding of secreted full-Anti–PD1 and scFv-Anti-PD1, culture supernatants from CAR T cells were collected on days 4, 7, 10, and 14 post-transfections. For full-Anti-PD1 detection, human PD-1/PDCD1 Protein, His-tag (ACRO Biosystems, cat# PD1-H522a) was incubated overnight at 4 °C at a total concentration of 50 ng per well in 100 µL of 1xPBS. The next day, plates were washed three times with PBS containing 0.05% Tween-20 (PBST) and incubated with 200 µL of culture supernatants, followed by two-fold serial dilutions at 37 °C for 1 h. After washing, wells were incubated with HRP-conjugated goat anti-human IgG Fc antibody (Invitrogen, cat# A55745; 1:8000 dilution in PBST) for 1 h at 37 °C. Following additional washes, 100 µL of SIGMAFAST™ OPD substrate (Sigma-Aldrich, St. Louis, MO, USA) was added and incubated for 20 min at room temperature in the dark. The reaction was stopped by adding 50 µL of 2 N H_2_SO_4_, and absorbance was measured at 492 nm using a microplate reader. This allowed quantitative determination of anti-PD1-Ig binding activity and concentration.

To detect the secretion and binding activity of anti-PD1 scFv, ELISA plates were coated overnight at 4 °C with recombinant human PD-1/PDCD1 protein, Fc tag, low endotoxin (MALS verified) (ACROBiosystems, Catalogue No. H5257-100 µg) at a concentration of 50 ng per well in 100 µL of 1xPBS (tested range: 10–100 ng/well). After washing and blocking with 5% skim milk in PBS-T, CAR T cell culture supernatants were added and incubated for 2 h at room temperature to allow binding of the secreted scFv to the immobilized PD1. Following washing, bound scFv was detected using an HRP-conjugated anti-His antibody (specific to the C-terminal His tag on the scFv) and developed with OPD substrate. The reaction was terminated with 2 N H_2_SO_4_, and absorbance was measured at 492 nm using a microplate reader.

### 4.7. Western Blot Analysis for Full Anti-PD-1 Antibody Secretion

The secretion of full Anti–PD1 was evaluated by Western blot. Equal volumes of culture supernatants (30 µL) were resolved on 10% SDS-PAGE gels, followed by protein transfer onto Immuno-Blot™ PVDF membranes (Bio-Rad, Hercules, CA, USA). Membranes were blocked with 5% skim milk in PBS to reduce nonspecific binding and subsequently washed with PBS containing 0.1% Tween-20 (PBST).

For detection, membranes were incubated with either HRP-conjugated goat anti-human IgG Fc antibody (Invitrogen, cat# A55745; 1:8000 dilution) or HRP-conjugated anti-His tag antibody (SouthernBiotech, cat# 4603-01L; 1:8000 dilution) in blocking buffer. After washing with PBST to remove unbound antibody, signals were developed using Clarity™ and Clarity Max™ ECL substrates (Bio-Rad). Protein bands corresponding to secreted full-anti–PD-1 were visualized by chemiluminescence, confirming antibody production by CAR T cells.

### 4.8. Cytotoxic Assay

Cytotoxicity was evaluated using Raji and Raji-PDL1 target cells at effector to target (E:T) ratios of 0.25:1, 0.5:1, and 1:1. Co-cultures were performed in 96-well plates and harvested at 24 h and 48 h time points. Following incubation, cells were collected, washed, and stained with anti-CD3 and anti-CD19 antibodies. 4′,6-diamidino-2-phenylindole (DAPI) dye was used for live/dead discrimination. Flow cytometry was then performed to assess target cell killing. The percentage of cytotoxicity was determined by comparing the proportion of viable target cells in co-cultures with effectors to that in target-only controls at the corresponding time points (24 h and 48 h). It was calculated by using the following formula: % Cytotoxicity = 100 − [(Number of live target cell count in experiment/No of total live target cell count in target alone)] × 100.

### 4.9. Rechallenge Cytotoxicity Assay

To assess serial killing capacity, a rechallenge assay was performed using Raji-PDL1 target cells in 24-well GREX plates (Wilson Wolf, St Paul, MN, USA) containing RPMI medium supplemented with 10% FBS and without cytokines. In the first round, 2 × 10^6^ effector T cells were co-cultured with 1 × 10^6^ Raji-PDL1 cells. After 3 days, cells were mixed thoroughly, and 100 μL of culture was collected for flow cytometry analysis, along with 10 μL for absolute cell counting. Fresh Raji-PDL1 cells (1 × 10^6^) were then added to initiate the second round of stimulation. This rechallenge process was repeated every three days for a total of four rounds. At the end of the assay, the total cells were harvested and stained with DAPI to distinguish live and dead populations. The remaining live cells were further stained with antibodies specific for CD3 (effector T cells) and CD19 (target cells). The absolute numbers of effector and target cells were quantified by flow cytometry.

### 4.10. Date Analysis

All DNA sequences were analyzed by Geneious Prime software version 2024.0.5. Schematic illustrations were made using Biorender. Data are presented as means ± SEM. Statistical analysis was performed using GraphPad Prism version 8 (GraphPad Software). Two-way ANOVA was used to determine statistical significance. *p*-values less than 0.05 were considered statistically significant.

## 5. Conclusions

In this study, we successfully generated a CD19 CAR capable of secreting either the full-length or scFv format of anti-PD1 antibody using a bidirectional promoter within a transposon-based system. Further in vivo evaluation in preclinical mouse models will be essential to validate the therapeutic benefits of this approach, particularly in sustaining effective secretion of the antibody formats. Importantly, the bidirectional promoter design offers a compact and efficient alternative to P2A or multi-vector strategies, ensuring balanced and reliable gene expression to enhance the therapeutic potential of CAR T cell therapy.

## Data Availability

All data relevant to the study are included in the article and uploaded as supplementary files.

## Supplementary Materials

The following supporting information can be downloaded at: https://www.mdpi.com/article/10.3390/ijms262311566/s1.
