# Supplementary material for: A Bidirectional EF1 Promoter System for Armoring CD19 CAR-T Cells with Secreted Anti-PD1 Antibodies
Source: Int J Mol Sci. 2025 Nov 28;26(23):11566. doi: 10.3390/ijms262311566 (PMC12692150; doi:10.3390/ijms262311566)
Supplement: Supplementary file 1 [file ijms-26-11566-s001.zip › Supplementary Figure S1.pdf]

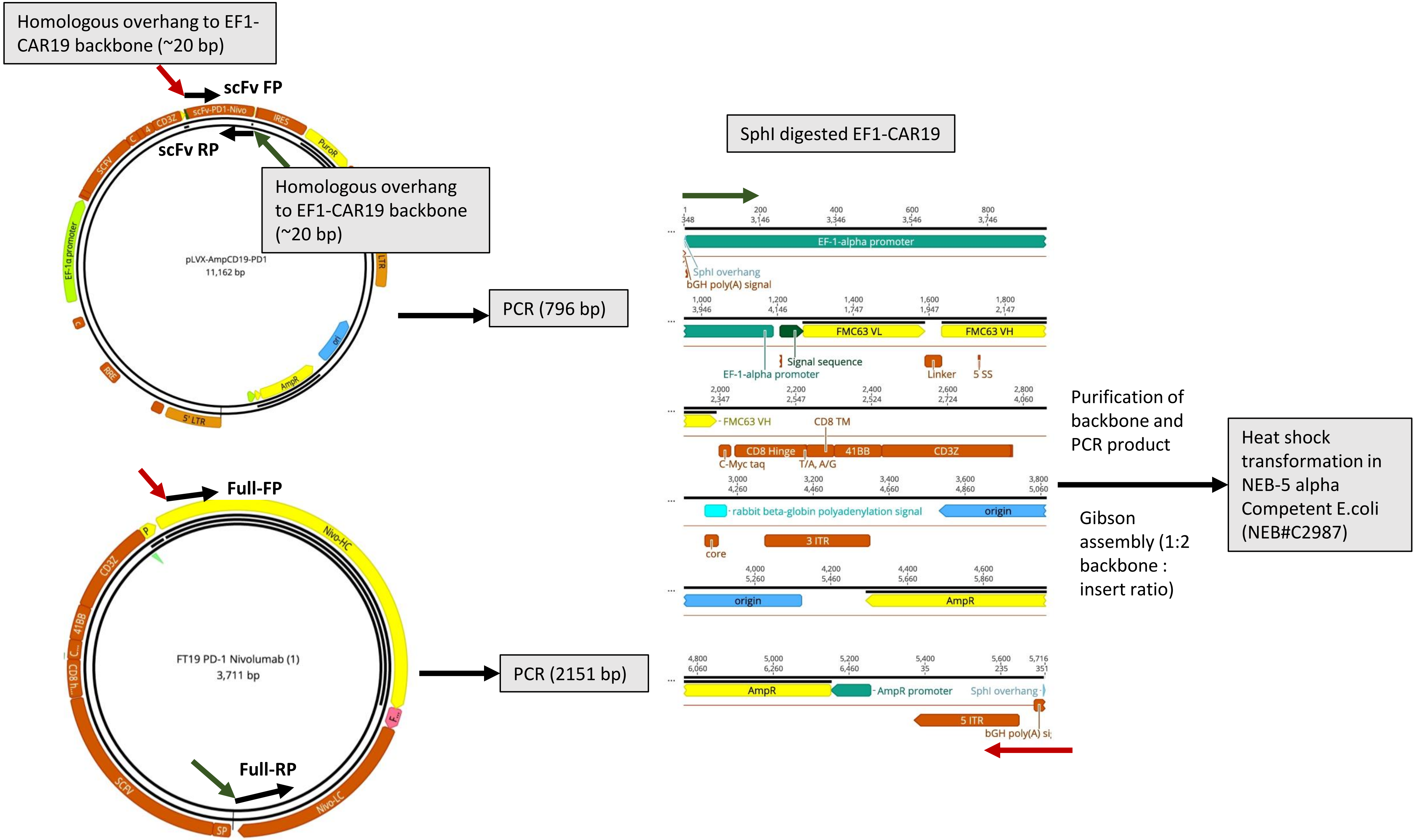
Homologous overhang to EF1-CAR19 backbone (~20 bp)
scFv FP
scFv RP
Homologous overhang to EF1-CAR19 backbone (~20 bp)
pLVX-AmpCD19-PD1
11,162 bp
scFv-PD1-Nivo
IRES
PuroR
CD3Z
SCFV
EF-1a promoter
LTR
ori
AmpR
RRE
5' LTR
PCR (796 bp)
Full-FP
Nivo-HC
CD3Z
41BB
SCFV
SP
Nivo-LC
FT19 PD-1 Nivolumab (1)
3,711 bp
Full-RP
PCR (2151 bp)
SphI digested EF1-CAR19
EF-1-alpha promoter
SphI overhang
bGH poly(A) signal
FMC63 VL
FMC63 VH
Signal sequence
EF-1-alpha promoter
Linker
5 SS
FMC63 VH
CD8 TM
CD8 Hinge
41BB
CD3Z
C-Myc taq
T/A, A/G
rabbit beta-globin polyadenylation signal
origin
3 ITR
core
origin
AmpR
AmpR
AmpR promoter
SphI overhang
5 ITR
Purification of backbone and PCR product
Gibson assembly (1:2 backbone : insert ratio)
Heat shock transformation in NEB-5 alpha Competent E.coli (NEB#C2987)

Final Construct

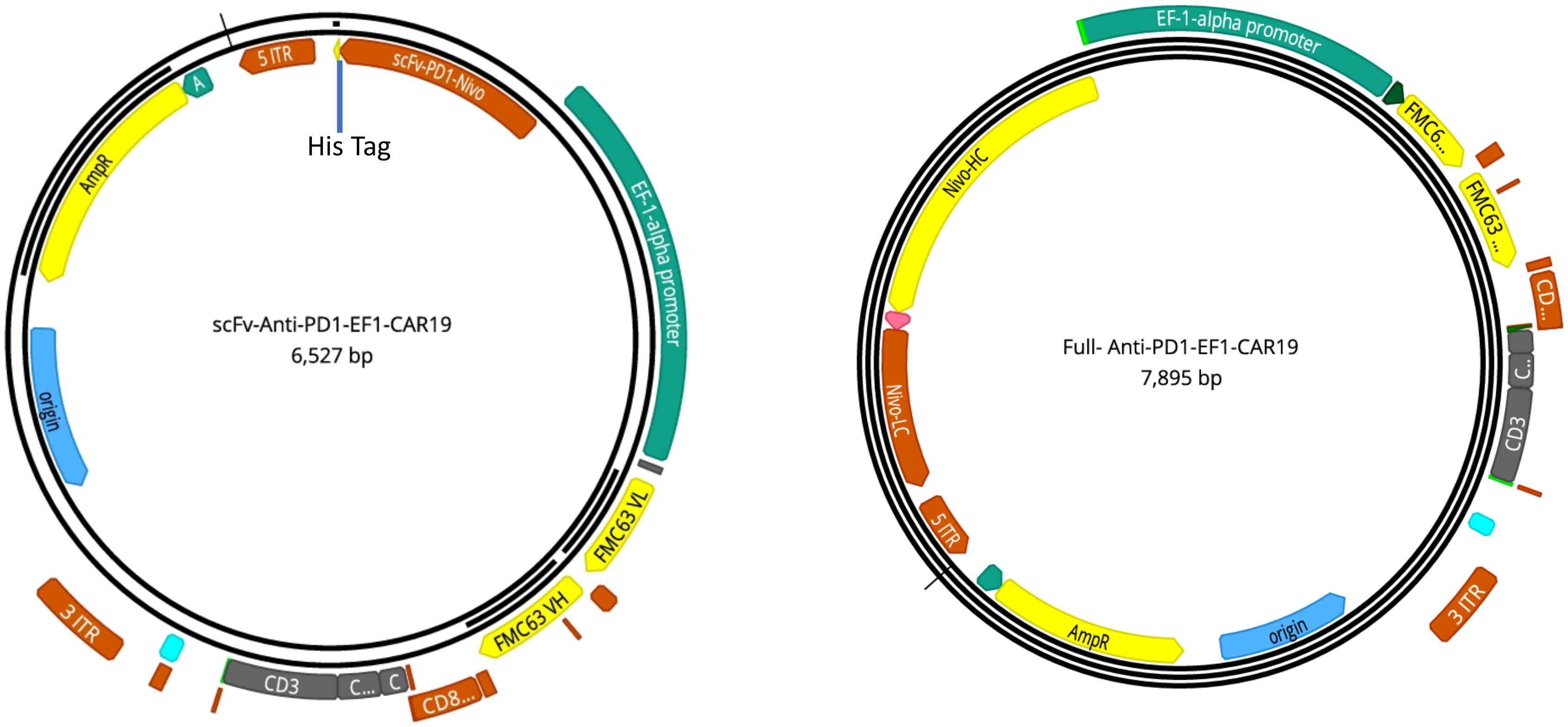

Supplementary figure S1. Construction workflow of the scFv-Anti-PD1 and full-length-Anti-PD1 expression plasmids. Schematic representation of the cloning strategy showing PCR amplification of the scFv (796 bp) and full-length antibody (2151 bp) inserts using primers containing ~20 bp homologous overhangs to the EF1-CAR19 backbone. The EF1-CAR19 plasmid was digested with *SphI* and purified along with the PCR products prior to Gibson Assembly (1:2 backbone-to-insert ratio). The assembled constructs were transformed into *E. coli* NEB 5-alpha competent cells, and positive clones were confirmed. Only the scFv construct contains a C-terminal His tag.
