# Supplementary material for: A Bidirectional EF1 Promoter System for Armoring CD19 CAR-T Cells with Secreted Anti-PD1 Antibodies
Source: Int J Mol Sci. 2025 Nov 28;26(23):11566. doi: 10.3390/ijms262311566 (PMC12692150; doi:10.3390/ijms262311566)
Supplement: Supplementary file 1 [file ijms-26-11566-s001.zip › Supplementary Figure S2.pdf]

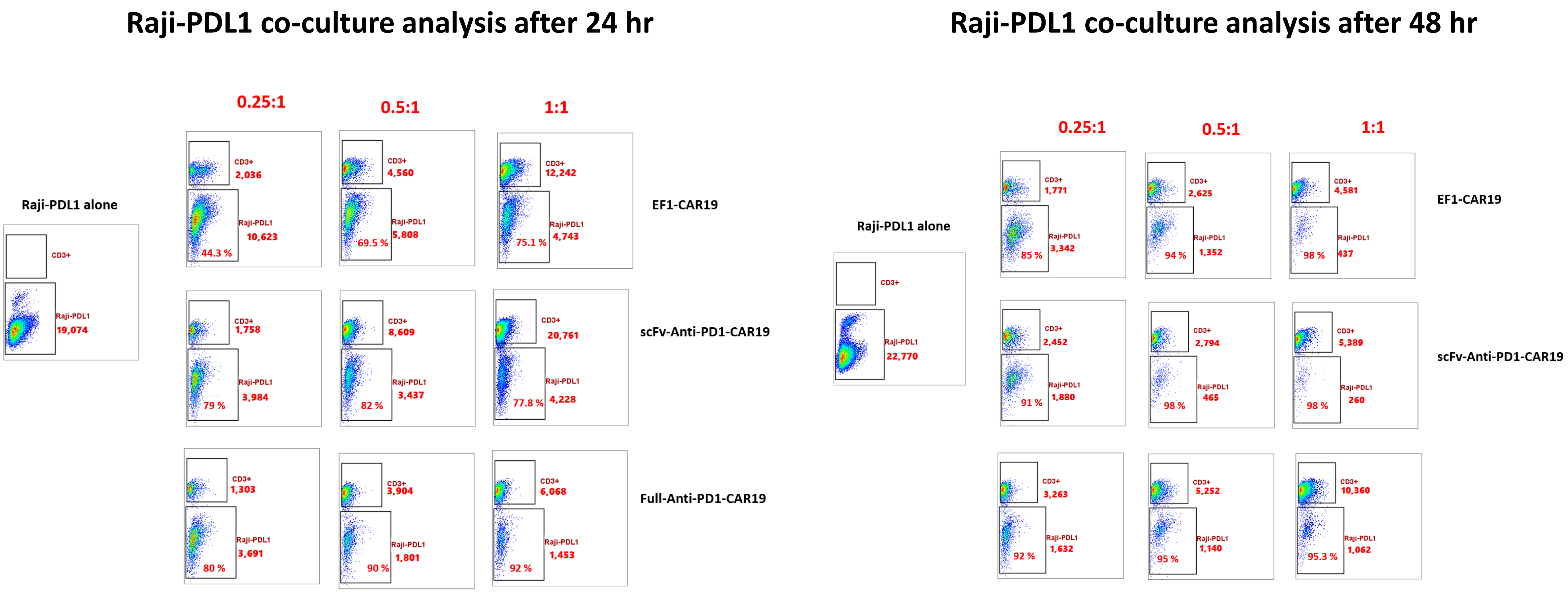

Supplementary figure 2. Raji-PDL1 co-culture assay. Representative flow-cytometry plots showing CAR T-cell functional response after 24 hr (left) and 48 hr (right) of co-culture with Raji-PDL1 target cells.
